# Tackling new psychoactive substances through metabolomics: UHPLC-HRMS study on natural and synthetic opioids in male and female murine models

**DOI:** 10.1038/s41598-024-60045-2

**Published:** 2024-04-24

**Authors:** Gaia Di Francesco, Camilla Montesano, Flaminia Vincenti, Sabrine Bilel, Giorgia Corli, Greta Petrella, Daniel Oscar Cicero, Adolfo Gregori, Matteo Marti, Manuel Sergi

**Affiliations:** 1 Department of Chemistry, University La Sapienza, 00185 Rome, Italy; 2 Department of Translational Medicine, Section of Legal Medicine and LTTA Centre, University of Ferrara, Ferrara, Italy; 3 Department of Chemical Sciences and Technologies, University of Rome “Tor Vergata”, 00133 Rome, Italy; 4 Carabinieri, Department of Scientific Investigation (RIS), 00191 Rome, Italy; 5 Department of Anti-Drug Policies, Collaborative Center for the Italian National Early Warning System, Presidency of the Council of Ministers, Rome, Italy

**Keywords:** Metabolomics, Analytical chemistry, Mass spectrometry

## Abstract

Novel psychoactive substances (NPS) represent a broad class of drugs new to the illicit market that often allow passing drug-screening tests. They are characterized by a variety of structures, rapid transience on the drug scene and mostly unknown metabolic profiles, thus creating an ever-changing scenario with evolving analytical targets. The present study aims at developing an indirect screening strategy for NPS monitoring, and specifically for new synthetic opioids (NSOs), based on assessing changes in endogenous urinary metabolite levels as a consequence of the systemic response following their intake. The experimental design involved in-vivo mice models: 16 animals of both sex received a single administration of morphine or fentanyl. Urine was collected before and after administration at different time points; the samples were then analysed with an untargeted metabolomics LC-HRMS workflow. According to our results, the intake of opioids resulted in an elevated energy demand, that was more pronounced on male animals, as evidenced by the increase in medium and long chain acylcarnitines levels. It was also shown that opioid administration disrupted the pathways related to catecholamines biosynthesis. The observed alterations were common to both morphine and fentanyl: this evidence indicate that they are not related to the chemical structure of the drug, but rather on the drug class. The proposed strategy may reinforce existing NPS screening approaches, by identifying indirect markers of drug assumption.

## Introduction

New psychoactive substances (NPS) are a complex and diversified group of drugs, mainly of synthetic origin^1^, with pharmaco-toxicological properties that emulate the traditional drugs of abuse.

NPSs are not under control by the United Nations Drug Conventions but they have the potential to become a danger for public health comparable or even higher than the traditional drugs^2^. Despite being classified as drugs of abuse, NPS still may escape consistent legislation; this is mainly due to their complexity and the rate at which they are released in the global market each year, hampering their detection in seizures and biological samples^3^. These designer drugs are being subdivided into six main categories in relation to their effects: stimulants, synthetic cannabinoid receptor agonists, hallucinogens, dissociatives, sedatives and novel synthetic opioids (NSOs)^4^. This latter class is among the most dangerous and fastest-growing group worldwide; from 2009 about 57 NSOs were identified in the European market^5^. It has been estimated that in 2020, among 68,630 opioid-related deaths recorded, 56,516 involved NSOs^6^. Nowadays the main NSOs include fentanyl derivatives (such as acetylfentanyl, carfentanyl) and non-fentanyl opioids like isotonitazene and brorphine^7^.

Due to the enormous number of substances synthesised every year, the complexity and variety of chemical structures of NSOs, the lack of analytical standards, and the unknown metabolic pathways by which they are excreted, identifying and quantifying NSOs, or more generally NPS, in biological fluids takes on a high degree of complexity. The target methods, typically based on low resolution mass spectrometry (LRMS), are not able to reveal NPS, which may present slight modifications respect to known molecules, nor to identify their metabolites; therefore, target methods require frequent database updates to include new substances, followed by subsequent re-validation of analytical methods. In order to mitigate these issues, untargeted high-resolution MS (HRMS) represents an alternative approach for the development of new screening strategies; in fact, untargeted methods, have the potential to not be tied to the chemical structure of the compounds and are then more suitable for NPS analysis^8^. Specific mass spectrometric approaches have been recently identified as valuable strategies for some NPS class detection^9,10^, but they generally rely on structure similarity to known molecules.

In this context, metabolomics-driven approaches that do not directly focus on the chemical structure of the drugs but rather on the impact of NPS on the metabolome^11,12^ may be successful. Additionally, by identifying specific metabolic fingerprints related to drug consumption, more insights into the mechanisms by which the drug acts can be drawn and it may be helpful to underline metabolic perturbations associated with drug addiction. A few studies have already demonstrated the applicability of this strategy for illicit drugs investigations, including, for example, GHB^13^, MDMA^14^, and synthetic cannabinoids^15^. However, changes observed on the endogenous level appear relatively small and unspecific, suggesting that more well-designed studies in animals are strongly needed^11^.

Metabolomics can be undertaken through targeted and untargeted methods; in this last case the objective is to characterize as many metabolites as possible (often more than 10,000 features). Currently, the state-of-the-art technologies employed in untargeted metabolomics are nuclear magnetic resonance (NMR) and mass spectrometry (MS), often coupled with separation techniques.

Liquid chromatography coupled to high resolution mass spectrometry (LC-HRMS) is often used to detect hundreds or thousands of metabolites in a single sample in a highly sensitive and reproducible manner^16^.

The goal of this study was to identify characteristic metabolic fingerprints arising from the consumption of traditional and synthetic opioids. This class of drugs was selected considering the high number of available substances with diversified structures along with the recent rise in acute intoxication and overdose deaths linked to NSOs. These molecules are particularly difficult to identify in biological matrices, posing a difficult challenge for forensic chemists. This is mainly due to three aspects: (i) the variety of substances in the market and the speed with which new NSOs are introduced; (ii) the rapidity with which opioids are transformed into their metabolites; (iii) the low concentrations in biological fluids mainly due to the high psychoactive and analgesic potency of the substances even in low doses^17^. The development of alternative strategies that can counteract the illicit spread of NSOs is, therefore, of great interest. The experimental design involved in-vivo mice models: several mice, both males and females received a single administration of vehicle, morphine or fentanyl. Clinical effects were initially investigated to evaluate the doses of the two drugs that induced similar effects. Urine samples, collected before and after administration, at different time points, were then analyzed by LC-HRMS, with a robust untargeted metabolomics workflow. Finally, multivariate statistical classification methods were applied to the obtained datasets to recognize specific alterations in the endogenous metabolite pathways, connected to opioid consumption.

## Results and discussion

### Study of the clinical effects induced by morphine and fentanyl

In order to be able to compare the two opioids by metabolomic analysis, we used a dose of morphine (30 mg/kg) and fentanyl (6 mg/kg) capable of causing the same pharmaco-toxicological effects both as analgesia and respiratory depression in male and female mice. These doses were chosen based on previous studies in CD-1 male mice^18,19^; through several tests the equivalence of these doses was demonstrated.

**Figure 1:**
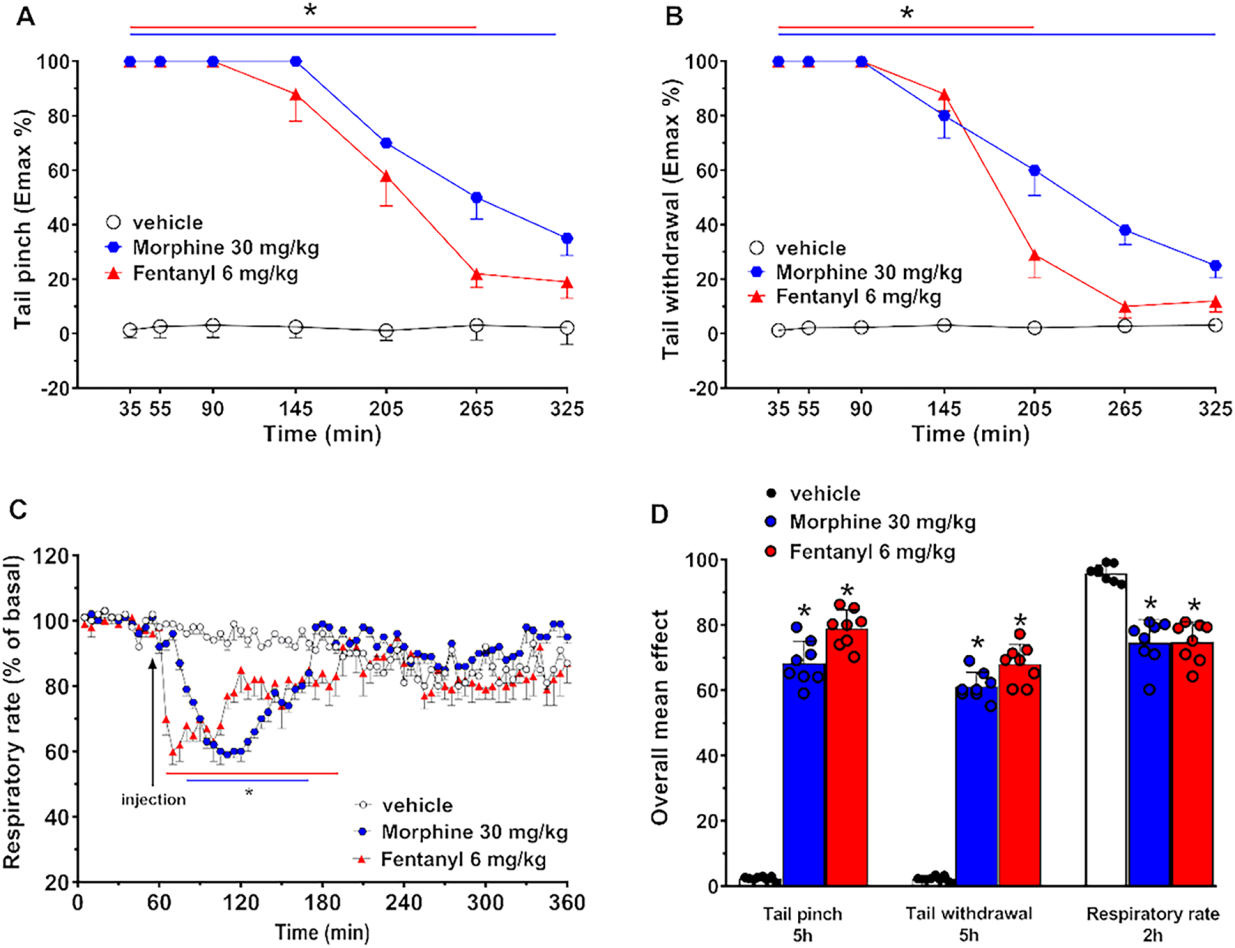
Effect of the systemic administration of morphine (30 mg/kg i.p.) and fentanyl (6 mg/kg i.p.) on the tail pinch test (**A**), tail withdrawal (**B**), respiratory rate (**C**) of the mouse. Overall mean effects of both opioids on mechanical (5 h mean effect) and thermal (5 h mean effect) analgesia and respiratory rate (2 h mean effect) were also reported (**D**). Data are expressed as percentage of maximum effect (see “Materials and methods”) and represent the mean ± SEM of eight determinations for each treatment. Statistical analysis was performed by two-way ANOVA followed by the Bonferroni’s test for multiple comparisons. *p < 0.05, versus vehicle.

Systemic administration of morphine (30 mg/kg) and fentanyl (6 mg/kg) equally increased the threshold to acute mechanical pain stimulus in the tail pinch test (Fig. 1A, D). The mechanical analgesia was significantly affected by treatment [F(2, 147) = 484.8, p < 0.0001], time [F(6, 147) = 54.05, p < 0.0001] and time × treatment interaction [F(12, 147) = 14.65, p < 0.0001].

Another observation was that the differential doses of the two opioids equally increased the threshold to acute thermal pain stimulus in the tail withdrawal test (Fig. 1B, D). The thermal analgesia was significantly affected by treatment [F(2, 147) = 590.1, p < 0.0001], time [F(6, 147) = 107.5, p < 0.0001] and time × treatment interaction [F(12, 147) = 31.16, p < 0.0001]. Morphine (30 mg/kg) or fentanyl (6 mg/kg) induces the same mechanical and analgesic effect on male and female mice (data not reported). The analgesic effects of morphine and fentanyl were previously investigated demonstrating the central role of the mu opioids receptors in the analgesia effects of opioids^18,19^.

In addition, morphine (30 mg/kg) and fentanyl (6 mg/kg) equally reduce the respiratory rate in mice (Fig. 1C, D). The respiratory rate was significantly reduced by treatment [F(2, 1512) = 81.32, p < 0.0001], time [F(71, 1512) = 11,20, p < 0.0001] and time × treatment interaction [F(142, 1512) = 5 650, p < 0.0001]. Finally, the tested doses of the two opioids induces the same inhibition of respiratory rate in male and female mice (data not reported). Respiratory depression is a common adverse effect observed with many opioids. The effect of morphine and fentanyl on this parameter is well-established demonstrating a central role of the mu opioid receptors on this negative effect. In addition, in case of respiratory depression induced by fentanyl (6 mg/kg, i.p.), naloxone (mu opioid receptor antagonist) redosing was necessary to block this impairment^18^.

### Metabolomic study design

**Figure 2:**
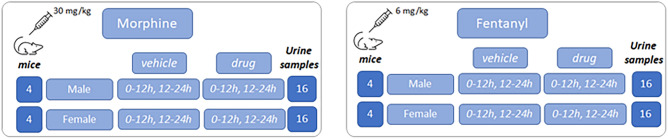
Schematic representation of the animal groups, outlining the number of samples collected.

In this work we studied the alteration of the urinary metabolic profile of 16 female and male mice (CD-1^®^) treated with two opioids: morphine (a natural opioid) and fentanyl (a synthetic opioid) as summarized in Fig. 2. These two substances were selected because their therapeutically use and their pharmacological effects have been extensively studied. Furthermore, the pharmacological effect that they exert on the central nervous system is known, and is common to most opioids (synthetic and natural)^20^ allowing to focus on common metabolic changes induced by their administration.

Reliable detection of drug influence on the metabolome, requires standardized conditions to exclude confounding factors and in this context, animal research provides a degree of experimental control and precision not usually feasible in studies using human subjects.

Different doses of the drugs were administered (30 mg/kg for morphine and 6 mg/kg for fentanyl) but they can be considered as “equivalent” regarding the main opioid effects on the threshold to thermal and mechanical pain and respiratory activity, as discussed in the previous paragraph. Moreover, these effects overlap in male and female mice.

### Analytical strategy and data quality

Two different chromatographic systems, reverse phase (RP) and hydrophilic interaction liquid chromatography (HILIC), both in positive and negative ionisation mode, were featured to achieve a maximum coverage of the entire metabolome. In addition, all samples were analysed in random order to avoid systematic effects on the instrument performance during the batch run.

Considering the high number of samples analysed within the study, robust quality assurance of the LC-HRMS data was crucial. Pooled QCs samples were injected every six samples to provide signal drift correction for each metabolic feature detected; signal correction was performed by Compound Discoverer software which provided normalized areas.

Internal standard (ISWS-A and ISWS-B) were spiked into all samples and QCs at equal concentration; samples were quantitatively processed before data analysis and IS peak area was calculated in order to verify the variability due to sample processing or any analytical platform operation. IS peak areas and Rt were used to create Shewhart control charts in excel. Median area values were calculated, and it was verified that IS area and Rt were within median ± 2σ for each analysed sample. No outliers were found in the analysed batch and all samples were then maintained for the following statistical evaluation.

#### Post-acquisition probabilistic quotient normalization (PQN)

Urine normalization is crucial to account for variations in the overall concentration of metabolites caused by different dilutions. Dilution is a significant variation that affects the concentration of all metabolites by the same factor, it generally derives from water consumption and other physiological factors. On the other hand, metabolomic responses mainly affect some metabolites in body fluids and, consequently, only some peaks of the corresponding spectrum. These specific changes are visible as relative changes in the concentrations of a few metabolites relative to the concentrations of all other metabolites, representing the overall concentration of the sample. Before the normalization, the difference associated with several hydration profile could hide the variation of metabolites concentrations between distinct samples. Different normalization techniques, which can be used either pre-acquisition (preventive) or post-acquisition (curative), are commonly applied in LC-HRMS studies. Among the existing normalization approaches, PQN is a statistical method belonging to a data-based driven approach and is also demonstrated that PQN reduce the impact of concentration variability^21^. In general all standardization strategies performed equally at level of signal variance^22^ but just two, including PQN, maintained the highest level of performance at recovering peak intensities. This normalisation method considers that changes in a single metabolite concentration does not affect the final data, while changes in sample concentration (which corresponds to different hydration) determines alteration in the entire chromatogram. This algorithm is based on five steps: (i) perform an internal normalisation (use of internal standards); (ii) create a reference spectrum by calculating the median of each variable across all samples; (iii) calculate the quotient of all variables of interest in the chromatogram by dividing the initial concentration (or intensity in the case of MS data) by the median of each variable; (iv) calculate the median of these quotients; (v) divide the initial concentrations of each spectrum by the quotient calculated in the previous step. The choice of this post-acquisition strategy allowed a simple manipulation of the samples which were all diluted by the same factor before LC-MS/MS analysis. Urine was extensively diluted (dilution factor = 20) to avoid dissimilar matrix effects in the analysed samples as also confirmed by the ISs which had similar response in all the analysed samples.

#### Datasets acquisition and filtering

Following MS raw data acquisition, each batch was loaded into Compound Discoverer^™^ software separately for peak picking, alignment, data deconvolution and normalization to the pooled QC, resulting in four tables for each chromatographic separation and polarity. A higher number of features was detected in RP (both positive and negative ion mode) compared to HILIC conditions. The original HILIC and RP datasets included approximately 15,000 and 23,000 metabolic features, respectively (total 37,000 hits when the four datasets were combined).

At that stage, PQN post-acquisition normalization of urine samples was carried out. Before implementing the filtration strategies, to keep only the information about the endogenous metabolites, all the hits belonging to morphine, fentanyl and their metabolites such as morphine-3-glucuronide, hydromorphone, norfentanyl, methoxyacetyl-norfentanil, β-hydroxyfentanyl and ω-hydroxynorphentanyl were removed from the MS data matrix. These features were identified based on their exact mass, isotopic pattern and MS/MS spectra.

Thus, two filtering processes were serially applied to reduce redundancy and noise. The first data reduction approach aimed at excluding from the dataset the noisy features; the rationale of this dilution/filtration strategy is that a linear relationship between MS signal intensity and metabolite concentration should exist for the features that are useful to characterise metabolic variations in the biological samples^23^. Diluted QCs were prepared to distinguish between informative and uninformative signals; for each metabolic feature the correlation factor between the peak area and the dilution factor was calculated. Only the features which showed a coefficient > 0.8 were maintained in the dataset while the others were filtered out; these signals are likely to occur in case of ionization competition, saturation effects, contamination from the solvents, column leak etc. Nearly 64% features were filtered out at this stage. Clustering of multiple ion types and fragments was then attempted by Pearson’s correlation of signal intensities^24^ by exploiting specific Excel functions. In this script HILIC and RP features were processed separately; features were listed based on their retention time (Rt) and a matrix correlating each couple of features that shared the same retention time was created. Accordingly, features with the same Rt and a r > 0.8 were grouped together. Inside each group only the metabolic features with the highest intensity were maintained for the following statistical analysis. Actually, new datasets were built combining the non-correlated hits with the hits showing the highest intensity among the correlated ones. The new datasets consisted of 8119 (HILIC) and 11,211 (RP) features (total 19,330 hits). The filtered dataset was imported to SIMCA^®^ for statistical analysis.

### Multivariate analysis and identification of relevant features

Statistical analyses were performed using SIMCA^®^ (version 17.0, Sartorius, Göttingen, Germany). Pareto scaling was used since it augments the representation of the low concentration metabolites by dividing each variable by the square root of the standard deviation of the variable, without increasing the noise contribution to the model^25^. Normally PCA is the first approach allowing an identification of difference, or similarities, between samples without a prior knowledge of sample class. Instead, OPLS-DA, is used to maximize the separation between sample classes, focusing on extracting the significant variable for group separation. According to the multivariate analysis, the variables that mostly contribute to the classification can be recognized as the significantly changed metabolites.

**Figure 3:**
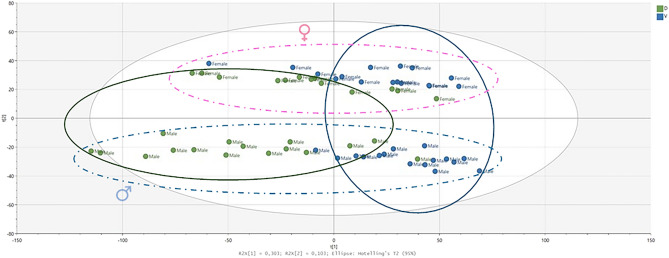
PCA scores plot of the filtered data set (RP and HILIC pos and neg) displaying the partial separation between drug-treated (green) and vehicle-treated mice (blue). Male animals also showed separation from females as highlighted by the dotted lines.

Initially, principal components analysis (PCA) was used as an unsupervised multivariate analysis method to visualize the data, eliminating possible outliers and controlling instrumental drift during the analysis. The PCA obtained for controls and treated animal urine samples is shown in Fig. 3. The score plot showed that drug-treated (in green) and vehicle-treated (in blue) mice formed two distinct clusters in accordance with the purpose of the study, and in addition it was possible to observe a difference between male and female animals. Contrarily, morphine and fentanyl treated samples were not separated in the PCA score plot, indicating that the metabolic signature upon administration of both opiates is similar.

In general, the analysis showed no outliers neither batch effect.

#### Changes in urine metabolites of mice administered with morphine and fentanyl

**Figure 4:**
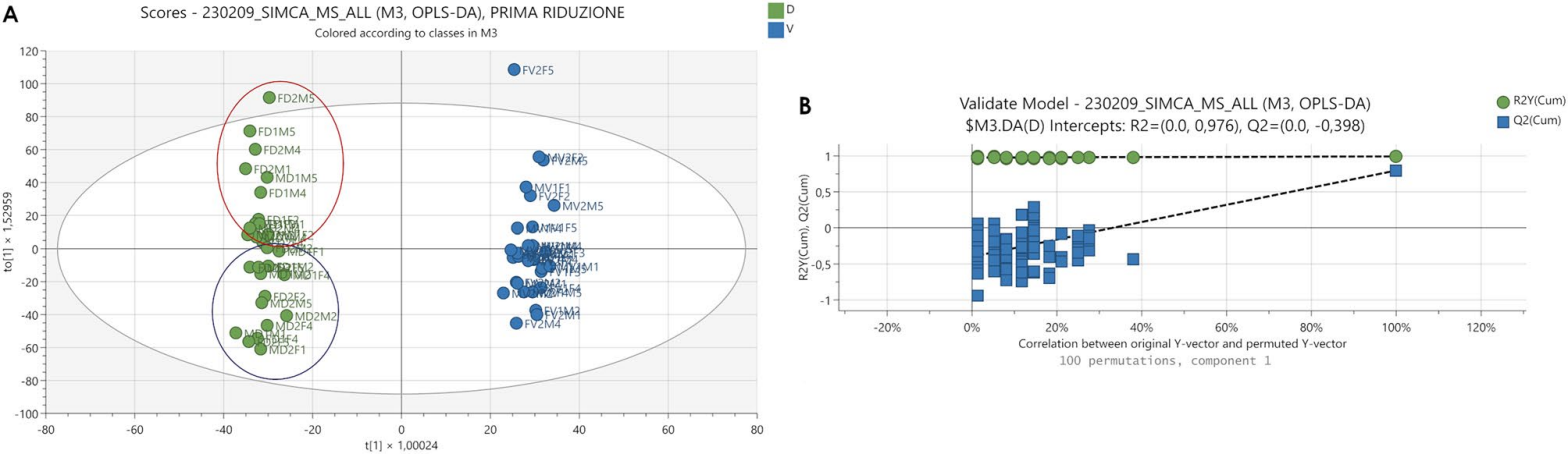
(**A**) OPLS-DA scores plot displaying the separation for drug-treated (green) and vehicle treated (blue) mice. (**B**) Permutation test showing the goodness of the model built.

Morphine is a natural opioid while fentanyl is a synthetic molecule; they both bind to the opioid receptors in the brain, so that their intake is expected to induce changes at molecular, biochemical, and neural system levels. OPLS-DA was used to discriminate between vehicle and drug-treated animals. For this model, R^2^Y (cumulative), Q^2^ (cumulative) and CV-Anova (p-value) were 0.979, 0.797 and 1.58 × 10^−11^ respectively, confirming the validity of the model. In addition, to rule out the non-randomness of separation between groups, a 100-iteration permutation test was performed and the result, showed in Fig. 4, further confirmed that the model was valid.

Figure 4 also displays the OPLS-DA scores plot for vehicle vs. treated animals. It should be noticed that in the drug-treated group, except for a few outliers, fentanyl and morphine treated mice formed two distinct clusters, while collection time did not affect sample distribution.

Possible biomarkers were identified by extrapolating the variables of importance in projection (VIP), i.e. those compounds that maximise group separation. OPLS-DA returned nearly 100 potentially interesting features with a VIP > 1.5, that were then tentatively elucidated in relation to their accurate mass, isotopic pattern, retention time and MS/MS spectra matching with accessible database (GNPS, HMDB, mzCloud, ChemSpider). In total, 10 metabolites could be putatively identified with a confidence level of two according to the metabolomics standard initiative (MSI) and they are summarized in Table 1. Other significant features were failed to be annotated to known metabolites, because several candidates could match to the observed MS peak and MS/MS information was not sufficient (or not available) to provide additional identification power. It can be observed that the differential metabolites were mainly involved in TCA cycle, tyrosine, phenylalanine and lipid metabolism.

**Table 1:** Altered metabolites in drug-treated versus vehicle-treated mice.

Mass	error (ppm)	Retention factor (K)	Formula	Putative annotation MS	Acquisition mode	mz Cloud match	Major fragment ions	Treated vs vehicle
117.0789	0.0	6.92	C_5_H_11_NO_2_	5-Amino-pentanoic acid	HC+	83.40%	69.03407 87.04449 100.0760 118.0863	↓
131.0696	0.9	0.24 (RP + /-) 8.36 (HC +)	C_4_H_9_N_3_O_2_	Creatine	RP+ /− , HC+	99.60%	87.0557 90.0553 132.0766	↑
89.0473	− 4.2	0.26	C_3_H_7_NO_2_	dl-Alanine	RP−	98%	60.9915 71.0122 89.9865	↑
132.0789	2.0	6.51	C_6_H_12_O_3_	5-Hydroxyhexanoic acid	RP+	73.80%	69.0704 73.0652 97.0650 115.0759	↓
174.0158	− 3.4	1.85	C_6_H_6_O_6_	Aconitic acid	RP−	95.50%	85.0279 115.0759 129.0179	↓
152.0473	-0.2	5.76	C_8_H_8_O_3_	p-Hydroxyphenylacetic acid	RP−	83.90%	93.0330 107.0487 121.0280 151.0745	↓
137.04772	− 0.3	8.34	C_7_H_7_NO_2_	Trigonelline	HC+	99.80%	94.0551 110.0603 138.0549	↓
183.0896	0.5	4.46	C_9_H_13_NO_3_	Epinephrine	HC+	80.4%	166.0974 140.0705 125.0471	↓
195.0896	0.5	0.31	C_10_H_13_NO_3_	n-acetyldopamine	HC+	71.3%	152.0796 135.0441	↓
195.0534	1.5	6.51	C_9_H_9_NO_4_	Dopaquinone	RP+	–	–	↓
230.1055	0.0	5.91	C_13_H_14_N_2_O_2_	Cyclic melatonin	RP+	–	214.0862 158.0964 84.0813	↓

Putative metabolites were identified by matching the accurate mass, isotope pattern or MS/MS data with mass spectral and compound libraries.

**Figure 5:**
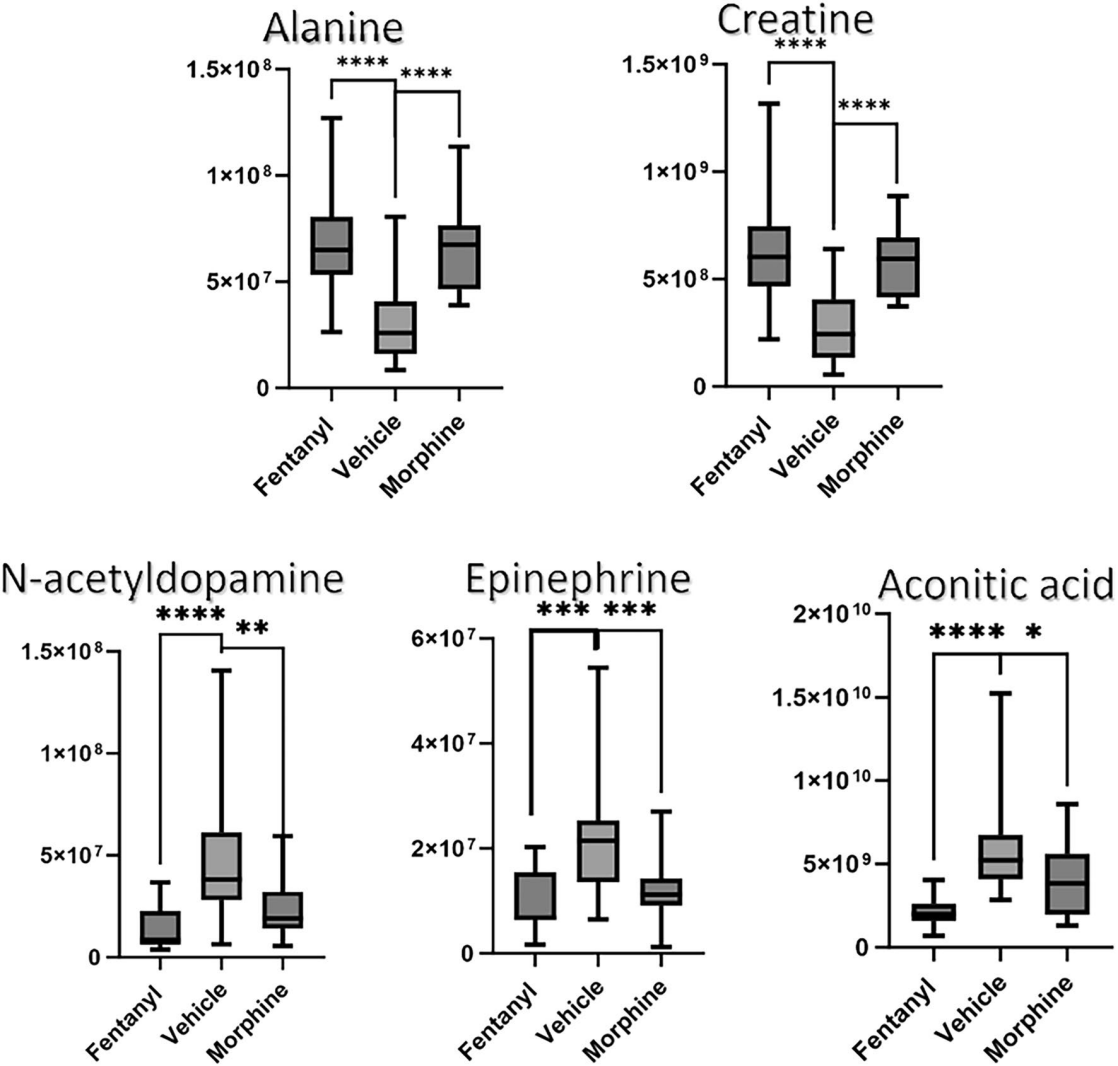
Boxplots of normalized area intensities of five among the most altered metabolites. p-value < 0.05*; < 0.01**; < 0.001***; < 0.0001****.

While aconitic acid, 5-amino-pentanoic acid, 5-hydroxyhexanoic acid and 3-phenylacetic acid mainly decreased in concentration compared to vehicle, creatine and alanine increased. In addition, it was observed that fentanyl affected the metabolome more severely than morphine, normalized areas for the most altered metabolites are exemplarily depicted in Fig. 5.

These observations were generally in agreement with previous studies that explored the alterations in the human or animal metabolome following opioid consumption (mainly morphine or heroin) to shed light on their mechanism and toxicity^26^. In most cases the metabolic signature of opioids has been studied on the brain and it has been observed that morphine and heroin lead to profound alterations in the neurotransmitters levels as well as energy and amino acid metabolism^27^; in addition significant disturbances in the glutamine–glutamate–GABA (Gln–Glu–GABA) axis were a common finding^28–30^. Fewer studies investigated the biomolecular perturbations elicited by opioids in peripheral biofluids with translational value (e.g., blood, urine), which may allow for the identification of biomarkers of opioid assumption. Zaitsu et al. investigated the metabolic profiling of urine and blood plasma in rat models of drug addiction and found that TCA cycle intermediates significantly changed in the urine of morphine addicted rats, while 3-hydroxybutyric acid, l-tryptophan, cystine levels significantly decreased in plasma^31^. Similarly, Zheng et al. examined the metabolic phenotype of rats exposed to heroin and observed that the drug induced an acceleration of the TCA cycle and the metabolism of free fatty acids with decreased tryptophan and 5-hydroxytryptamine levels in peripheral serum but increased urinary tryptophan and 5-hydroxyindoleacetate^32^. In a more recent study, involving heroin and morphine, it was observed that both drugs interfered with lipid metabolism, tricarboxylic acid (TCA) cycle and amino acid metabolism; in this case the effects of morphine on metabolites in urine were somewhat dissimilar to those of heroin except for aconitic acid, cysteine, glycine, and oxalic acid, which significantly decreased with both drugs. It should be highlighted that in all these studies, metabolomics was exploited to investigate metabolite alterations in addicted subjects or after repeated administration so that the results of our study, which involved a single administration, may be somewhat different. The metabolic pathways, which were shown to be altered in our study, are similar to those observed by other authors, indicating that the metabolic signature upon opioid administration is generally independent from the dosage or the addiction status. Increased energy metabolism is common to drug abuse and was observed also following the intake of non-opioid drugs^26^, since neuronal activity is extremely energy demanding; numerous papers have revealed a disturbance in energy metabolism by drug abuse indicating an upregulation of the TCA cycle for increased energy metabolism and supply^33^. Aconitic acid is a key intermediate in the TCA cycle and has been implicated in the regulation of mitochondrial function and energy metabolism. The observed decrease in urinary aconitic acid by the two opioids is in agreement with the study of Lu et al.^34^ and suggest that both morphine and fentanyl accelerated energy metabolism. It is worth noting that aconitic acid reduction is higher in fentanyl respect to morphine. The observed distinction indicates that fentanyl possesses a higher level of energy metabolism compared to morphine. This correlation may be attributed to the increased lipophilicity and receptor affinity of fentanyl in comparison to morphine^35^. Other TCA cycle intermediates such as citric and ketoglutaric acid were putatively identified among the detected features, decrease of these metabolites following treatment was observed but the observed differences were not statistically relevant.

In parallel, creatine is thought to accelerate the recycling of adenosine triphosphate (ATP), the energy currency of the cell, to exert direct antioxidant effects through normalizing mitochondrial mutagenesis. Therefore, the increase of creatine levels may reflect a protective mechanism against energy exhaustion and oxidative stress^27^. On the other hand, alanine is involved in the glucose-l-alanine cycle, which plays a role in glycolysis and gluconeogenesis, another energy supplying pathway that can convert pyruvate into lactate and alanine. Up-regulation of glycolysis would lead to enhanced transamination of pyruvate to alanine, justifying the high levels of this amino acid in treated animals. A recent study by Alasmari et al.^36^, in mouse model of fentanyl overdose (50 µg/kg i.p) suggested the involvement of glucose-alanine cycle and gluconeogenesis in liver metabolism alterations associated to increased liver inflammation, after a treatment with a fatal dose of fentanyl. These findings suggest that the increasing amino acids in urine samples of mice treated with fentanyl and morphine could be related to a protective response against increased inflammations triggered by these opioids in different organs^37^.

Finally, similarly to previous studies, our results showed that opioids not only disrupt energy metabolism, but also the biosynthesis of catecholamines, which is connected to their metabolic effect on the brain. We observed a significant decrease of urinary levels of n-acetyldopamine, epinephrine and some of their metabolites.

Our observations are also in agreement with data obtained through proteomics, which indicated that the most frequently repeated proteins affected by morphine administration are enzymes crucial for mitochondrial function and involved in the metabolic energy pathways such as glycolysis/glucogenesis, TCA cycle, and oxidative phosphorylation (OXPHOS)^38^.

#### Sexual diergism in the metabolic signature upon administration of opioids

This study involved mice of both sex, because it is well known that the susceptibility to illicit drugs may vary considerably among males and females. Studies involving female mice are rather limited since researchers usually avoid using females because of their reproductive cycles and hormone fluctuations that may confound the study results. However, given that sex and gender deeply affect the subjective effects and pharmaco-toxicological responses to drugs^39^, it is of utmost importance to investigate sexual diergism in the metabolic changes associated with opioid use.

To this aim, to account for the expected and observed sex differences in the basal urine metabolome, each mouse was used as its own control: for each animal, the normalized peak area of each compound in the vehicle sample was subtracted from the normalized peak area after drug administration for both time points (i.e., 0–12 h and 12–24 h). The new dataset, obtained as previously described, was named *delta matrix*. In order to identify interesting features, the *delta matrix* was analysed by OPLS-DA.

**Figure 6:**
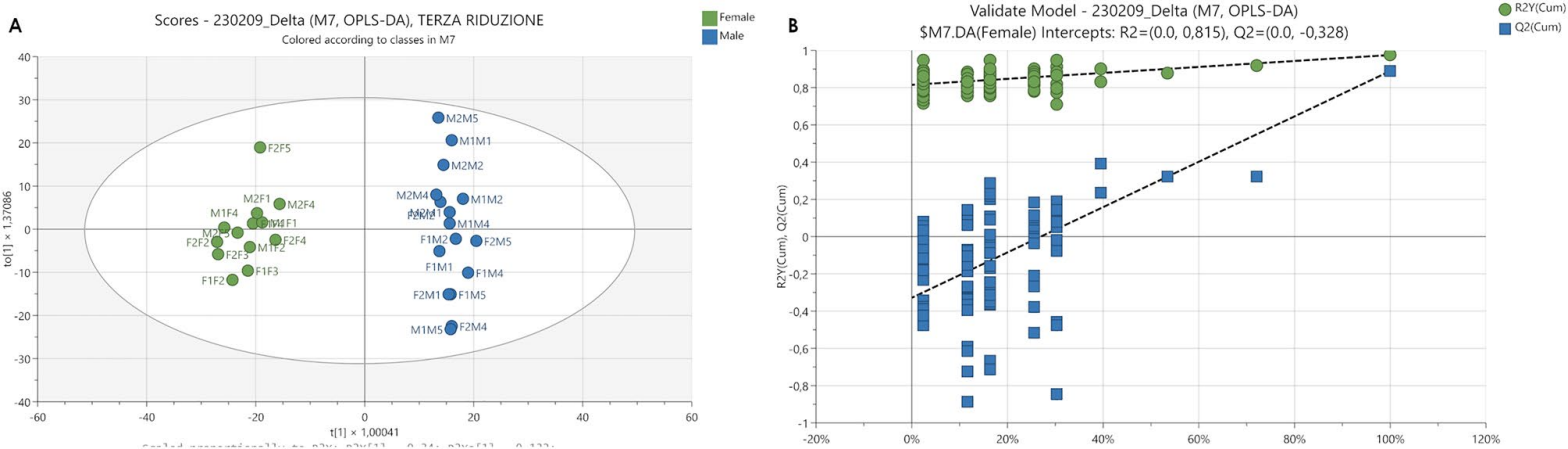
(**A**) OPLS-DA scores plot obtained with the delta matrix displaying the separation between female (green) and male (blue) mice. (**B**) Permutation test showing the goodness of the model built.

The resulting OPLS-DA scores plot of male vs female animals is shown in Fig. 6.

For this model, R^2^Y (cumulative), Q^2^ (cumulative) and CV-Anova (p-value) were 0.975, 0.889 and 1.97 × 10^−9^, respectively, confirming the validity of the model. Interesting features with a VIP > 1 were tentatively annotated with the same criteria previously described. Interestingly, it was observed that several significant features belonged to the compound classes of acyl-carnitines, amino acids and acetylated metabolites (Table 2).

**Table 2:** Altered metabolites in treated male versus treated female mice.

Mass	error (ppm)	Retention factor (K)	Formula	Putative annotation MS	Acquisition mode	mz Cloud match	Major fragment ions	Male vs vehicle	Female vs vehicle
188.0798	0.5	2.59	C_7_H_12_N_2_O_4_	n-Acetylglutamine	HC+	91.30%	84.0443 130.0492 172.0602	↓	=
203.1159	0.7	7.9	C_9_H_17_NO_4_	Acetylcarnitine	HC+	99.7%	85.0289 145.0539	↑	=
217.1315	0.4	7.06	C_10_H_19_NO_4_	Propanoylcarnitine	RP+	63.2%	182.1538 125.0961 76.0398	↑	=
231.1471	0.2	7.22	C_11_H_21_NO_4_	Butyryl carnitine	HC+	60.5%	173.081 85.0289 60.0815	↑	=
245.1627	− 0.1	9.74	C_12_H_23_NO_4_	Methylbutyryl carnitine	RP+	69.9%	85.0289	↑	=
259.1784	0.2	8.70 (RP +) 6.79 (HC +)	C_13_H_25_NO_4_	Hexanoylcarnitine	RP+ /HC+	97.7%	201.1120 85.0288 60.0814	↑	=
287.2097	0.1	6.46	C_15_H_29_NO_4_	Octanoylcarnitine	HC+	–	229.1436 85.0289 60.0815	↑	=
315.2410	0.3	9.27	C_17_H_33_NO_4_	Decanoylcarnitine	RP+	98.9%	267.1744 85.0289 60.0814	↑	=
272.1766	− 3.4	9.25	C_18_H_24_O_2_	17β-Estradiol	RP+	89.6%	255.1748 135.1162	↓	=
145.1104	0.8	0.67 (RP +) 7.64 (HILIC +)	C_7_H_15_NO_2_	Acetylcholine	RP+ /HILIC+	94.9%	87.0444 60.0814	↑	↓
268.1172	0.2	1.05	C_11_H_16_N_4_O_4_	n-Acetyl-l-carnosine	RP+	96.5%	156.0768 110.0716	↑	=
175.0634	0.6	9.86	C_10_H_9_NO_2_	Indole-3-acetic acid	RP+	99.3%	130.0650	=	↓
131.0948	1.3	2.02	C_6_H_13_NO_2_	Isoleucine	RP+	99.5%	86.0969 69.0705	↑	=
205.0774	0.7	4.03 (RP) 6.10 (HILIC)	C_8_H_15_NO_3_S	n-Propionylmethionine	RP+ /HILIC+	–	122.0271 117.0370 85.0653	↑	=
167.0444	0.5	4.96 (HILIC +) 0.7 (RP +)	C_5_H_5_N_5_O_2_	7,8-Dihydro-8-oxoguanine	RP+ /HILIC+	–	151.0752 126.0219	↑	=
173.1054	1.7	8.55 (HILIC +) 5.74 (RP +)	C_8_H_15_NO_3_	Hexanoylglycine	RP+ /HILIC+	82.5%	99.0808 76.0388 71.0861	↑	=
174.1005	0.3	9.01	C_7_H_14_N_2_O_3_	Acetyl-ornithine	HILIC+	72.7%	112.0759 70.0657	↑	=
214.1319	0.9	7.35 (RP +) 1.77 (HILIC +)	C_10_H_18_N_2_O_3_	Dethiobiotin	RP+ /HILIC+	67.4%	155.1068 137.0962 95.0860 57.0343	↑	=
216.1223	0.5	10.54	C_8_H_16_N_4_O_3_	n-α-Acetyl-l-arginine	HILIC+	86.3%	154.1225 130.0862 84.0813	↓	=
325.1014	1.5	4.87 (RP −) 1.02 (HILIC −)	C_11_H_19_NO_10_	n-Glycolylneuraminic acid	RP−/HILIC−	78.6%	263.0771 75.0072	↑	=

Putative metabolites were identified by matching the accurate mass, isotope pattern or MS/MS data with mass spectral and compound libraries.

**Figure 7:**
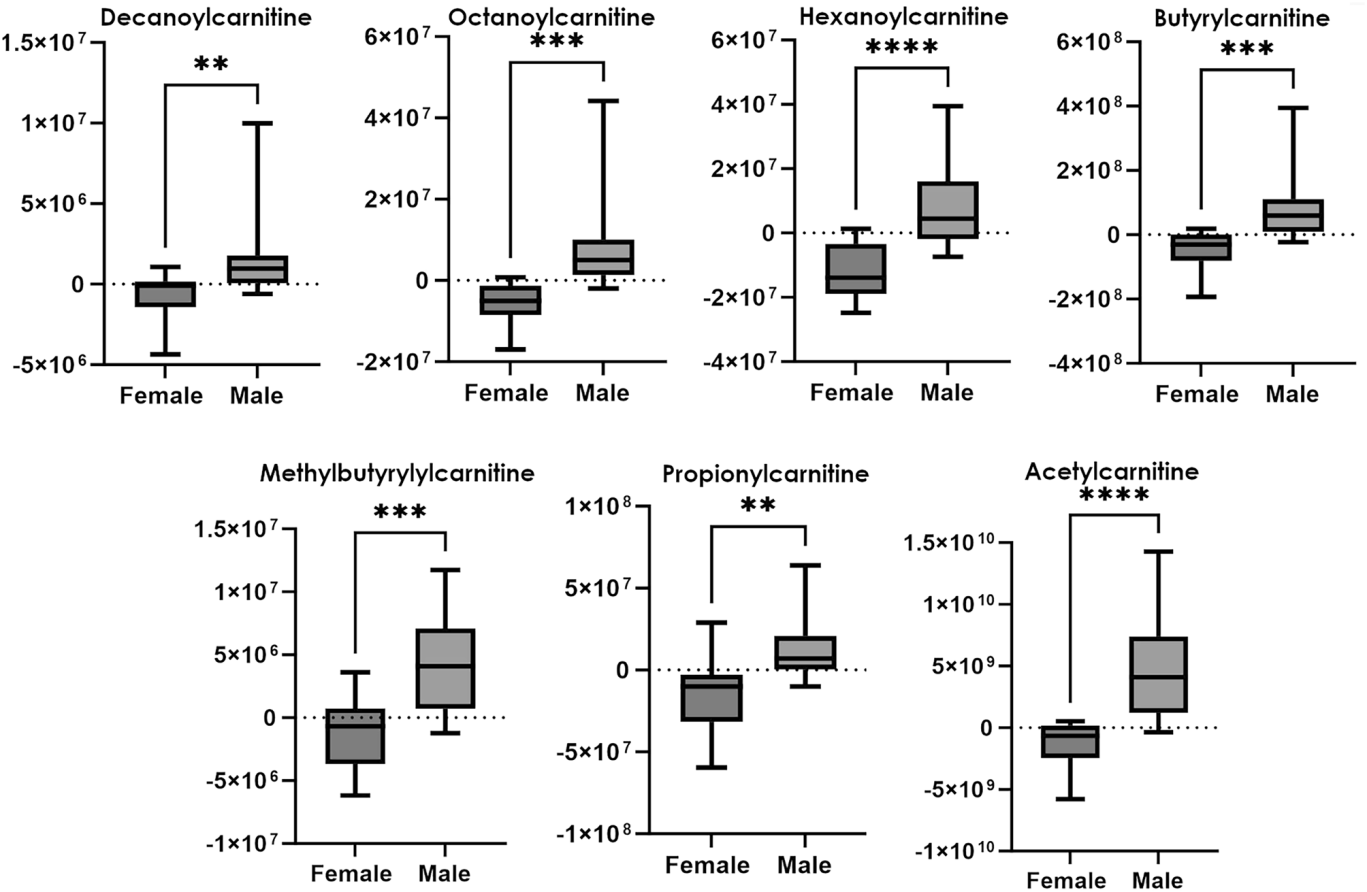
Boxplots of delta areas (obtained by substracting for every animal the normalized peak area of each compound in the vehicle sample from the normalized peak area after drug administration) for acylcarnitines. p-value < 0.05*; < 0.01**; < 0.001***; < 0.0001****.

For what concerns the former class, it was observed that short and medium chain acylcarnitines significantly increased for male mice after opioid intake (Fig. 7), especially when fentanyl was administered (data not shown). On the contrary, female mice exhibited stable levels of these metabolites or generally a slight decrease. Acylcarnitines play an essential role in regulating the balance of intracellular sugar and lipid metabolism. They serve as carriers to transport activated long-chain fatty acids into mitochondria for β-oxidation as a major source of energy for cell activities and they are also involved in maintaining the homeostasis of the mitochondrial acyl-CoA/CoA ratio. Moreover, acylcarnitines are involved in other physiological processes such as peroxidation of fatty acids, and production of ketone bodies. Therefore, their metabolism is not only related to the transport of fatty acids, but also plays a key role in regulating the balance of intracellular sugar and lipid metabolism^40^. Sexual diergism in acylcarnitine levels indicates a higher energy demand, associated with a higher oxidation of fatty acids, in male mice in response to opioid intake. An increase in medium and long chain acylcarnitines was also observed after consumption of stimulant drugs such as amphetamine and MDMA^14^. Interestingly, differences in energy metabolism related to sex were also reported by Leskanicova et al., who observed augmented acylcarnitine levels in male mice. Overall, the obtained data support evidence that opioids disruption in energy metabolism is exacerbated in male compared to female mice^41^.

Another interesting metabolite that was differentially altered between male and female mice is the neurotransmitter acetylcholine (ACh): it was significantly altered by opioid intake, especially for females, who exhibited a notable decrease of ACh urinary concentration after the treatment. Abnormalities in neurotransmitter metabolism following psychoactive drugs assumption is expected and has been reported also following cocaine assumption^26^, on the other hand anti-cholinergic properties of opioids are well known^42^. Interestingly, it was also observed that the cholinergic system appears to be more responsive to stress and other stimuli in female than in male mammals^43^ supporting the observed differences. It is noteworthy that ACh is the primary contractile neurotransmitter in mammal bladder detrusor^44^, so that the urinary retention phenomenon that was observed in treated female mice can be related to the low urinary ACh concentration detected in these animals.

Another remarkable altered metabolite is 7,8-dihydro-8-oxoguanine (8-oxoG) a recognized biomarker of cellular oxidative stress. 8-oxoG level increased only in male animals and may arise from the augmented rate of fatty acids β-oxidation, which is a well-known source of reactive oxygen species (ROS). Several studies have pointed to the relationship between opioid administration and increased production of ROS and it was also observed that oxidative stress status shows sexual diergism, with female rats generally exhibiting lower oxidative damage than males^45^. Several authors suggested that sex hormones and especially 17β-estradiol (E2) could be a major driver for these differences since they have been reported to directly regulate several mediators of the mitochondrial biogenesis program in several tissues^46^. Notably, a feature that resulted to be significantly altered in our dataset, can be putatively identified as E2, a significant decrease of this metabolite was observed in male drug-treated mice compared to vehicle-treated (see Table 2), supporting higher oxidative damage in these animals.

Opioids are known to severely affect the endocrine system and to depress the secretion of hormones at different levels of the hypothalamic–pituitary–gonadal axis^47^, sex differences are then expected for steroid levels following drug administration. Other significant features were putatively identified as belonging to the steroid class (based on exact masses, retention time and MS/MS fragments), however they could not be identified with suitable confidence due to the several isomeric forms of compounds in this class.

## Methods

### Chemical and reagents

Standards of morphine and fentanyl were purchased from Sigma–Aldrich (Burlington, MA). Morphine-d3, fentanyl-d5, EME-d3, XLR-11-d5, alprazolam-d5, used as internal standards (IS) were purchased from LGC (Milan, Italy).

All solvents and reagents used were LC-MS grade. Acetonitrile, methanol and formic acid 98–100% were from Merck (Darmstadt, DE). Ultrapure water for UPLC was produced by a Millipore Synergy UV water purification system (Millipore A/S, Copenhagen, DK).

### In vivo study

#### Animals’ behaviour and urine collection

Sixty-four CD-1 mice (CD-1^®^), 32 male and 32 female, were grouped in metabolic cages. Mice (48 for behavioural studies, 16 for urine collection) weighing 30–35 g (Centralized Preclinical Research Laboratory, University of Ferrara, Italy) were group housed (4/cage; floor area: 80 cm^2^/mouse; minimum enclosure height: 12 cm), exposed to a 12:12 h light–dark cycle (light on at 6:30AM) at a temperature of 20–22 °C and humidity of 45–55% and provided ad libitum access to food (diet 4RF25 GLP; Mucedola, Settimo Milanese, Milan, Italy) and water. Comparison of the effects induced by morphine and fentanyl was studied in groups of male and female mice from the same litter (i.e., siblings). All experimental protocols were in accordance with the U.K. Animals (scientific procedures) Act of 1986 and associated guidelines and the new European Communities Council Directive of September 2010 (2010/63/EU) and approved by the Italian Ministry of Health (license n. 223/2021-PR, CBCC2.46.EXT.21) and the Animal Welfare Body of the University of Ferrara. According to the ARRIVE guidelines, all possible efforts were made to minimise the animals’ pain and discomfort and to reduce the number of experimental subjects.

##### Behavioural studies

Mechanical and thermal analgesic effects of morphine (30 mg/kg) and fentanyl (6 mg/kg) were investigated using a battery of behavioural tests widely used in pharmacology safety studies for the preclinical characterization of new psychoactive substances in rodents^19,48–53^. All experiments were performed between 8:30AM and 2:00PM. Experiments were conducted blindly by trained observers working in pairs^54^.

##### Evaluation of pain induced by a mechanical and a thermal stimulus

Acute mechanical nociception was evaluated using the tail pinch test^48^. A special rigid probe connected to a digital dynamometer (ZP-50N, IMADA, Japan) was gently placed on the tail of the mouse (in the distal portion), and progressive pressure was applied. When the mouse flicked its tail, the pressure was stopped and the digital instrument recorded the maximum peak of weight supported (g/force). A cut off (500 g/force) was set to avoid tissue damage. The test was repeated three times and the final value was calculated by averaging the three obtained scores. Acute thermal nociception was evaluated using the tail withdrawal test^48^. The mouse was restrained in a dark plastic cylinder and half of its tail was dipped in 48 °C water; then, the length of time (in s) the tail was left in the water was recorded. A cut off (15 s) was set to avoid tissue damage. Acute mechanical and thermal nociception was measured at 0, 35, 55, 90, 145, 205, 265 and 325 min post injection.

##### Respiratory analysis

The experimental protocol to detect the respiratory activity used in this study is designed to monitor awake and freely moving animals with no invasive instruments and with minimal handling^55^. A collar was placed around the neck of the animal; this collar has a sensor that continuously detects the respiratory rate with a frequency of 15 Hz. While running the experiment, the mouse moves freely in the cage (with no access to food and water) monitored by the sensor collar using the software MouseOx Plus (STARR Life Sciences^®^ Corp. Oakmont, PA). In the first hour, a collar was placed around the animal’s neck to simulate the real one used in the test, thus minimising the possible effects of stress during the experiment. The real collar (with sensor) was then substituted, and baseline parameters were monitored for 60 min. Subsequently, the mice were given vehicle, morphine or fentanyl, by i.p. injection, and data was recorded for 5 h.

##### Data and statistical analysis

Antinociception (tail withdrawal and tail pinch tests) are calculated as the percent of maximal possible effect {EMax% = [(test − control latency)/ (cut off time − control)] × 100}. Data are expressed in Emax% (tail withdrawal and tail pinch), respiratory rate (expressed as respiratory rate per minute (rrpm). The statistical analysis of the effects of the individual substances in different doses over time and that of antagonism studies were performed using a two-way ANOVA followed by a Bonferroni test for multiple comparisons. The statistical analysis was performed using Prism software (GraphPad Prism, USA).

##### Urine collection

After identifying the “equivalent” doses of morphine (30 mg/kg i.p.) and fentanyl (6 mg/kg i.p.), a different group of 16 animals (eight male and eight female mice) were used in the urine collection study. Initially, all mice were given a dose of saline solution (0.9% NaCl), constituting the vehicle, by intraperitoneal injection; urine was cumulatively collected in the intervals 0–12 h 12–24 h after the administration. 24 h after vehicle administration the two groups were treated with 30 mg/kg of morphine and 6 mg/kg of fentanyl, respectively, through one-shot intraperitoneal injection; urine collection was carried out according to the protocol described above. Urine specimens were collected from mice individually placed inside metabolic cages (Ugo Basile SRL, Gemonio [VA], Italy) with free access to water and food^56–60^ in a colony room under constant temperature (23 °C–24 °C) and humidity (45–55%). Urine samples were collected in 2 mL tubes before drug injections (control), and every hour for 6 consecutive hours from the administration of the treatments [9,33]. After 6 h, urine was collected cumulatively in the 6–12, 12–24 and 24–36 h time interval and stored at − 20 ℃ until analysis.

### Analytical workflow

The proposed workflow for sample analysis and data quality assurance was based on an efficient metabolomics platform^61^, with some modifications. The detailed workflow description is reported below.

#### ISs solutions

Internal standard working solutions (IS-WS), containing morphine-d3, fentanyl-d5, EME-d3, XLR-11-d5, alprazolam-d5, were prepared by adding suitable volumes of the stock solutions to 20 mL of ultrapure water (ISWS-A) and acetonitrile (ISWS-B) respectively, in order to reach a final concentration of 20 ng mL^−1^ for all the standards. The solutions were maintained at − 20 °C.

#### Urine sample preparation

In order to minimise sample handling, the pre-treatment consisted of a sample dilution 1:10 (v:v) with a suitable solvent, chosen based on the chromatographic separation method, i.e. water for reverse phase (RP) and water:acetonitrile (50:50, v:v) for hydrophilic interaction liquid chromatography (HILIC). All samples were further diluted 2-folds with ISWS-A for RP analysis and ISWS-B for HILIC. Samples were vortexed and centrifuged (13,000* g*, 10 min); then the supernatant was transferred to a vial and randomised for LC-HRMS analyses.

#### Quality control samples and blanks

Two different types of QC samples were prepared and analysed along the batch: (i) pooled QCs prepared by mixing equal volumes (20 μL) from each sample (ii) dilution QCs prepared by diluting 2, 4 and 8 folds the pooled QC with water.

Pooled QCs were used for area correction in the subsequent data processing step, while dilution QCs were used to verify the linear response of the MS signal. QCs samples were diluted similarly to all samples, firstly 1:10 with water or acetonitrile:water (50:50, v:v) for RP and HILIC samples respectively and further with ISWS (1:1, v:v). Pooled QC samples (made by combining 5 µL of each urine sample) were injected first (n = 10) to condition the LC–MS system and obtain stable retention times and MS response. Subsequently, pooled QCs were injected every six true samples to perform intra-batch signal drift corrections. Dilution QCs were analyzed three times and were incorporated regularly along the sample list.

Blanks consisted of LC–MS grade water for RP analysis and AcN: water, 80:20 (v/v) for HILIC analysis; blank injection (n = 3) was performed at the beginning of the batch to collect a background signal to be excluded from the dataset.

#### UHPLC-HRMS conditions

##### HILIC and RP chromatography

Chromatography was performed using a UHPLC DionexTM UltiMateTM 3000 Rapid Separation Liquid Chromatography (RSLC) system (Thermo Fisher Scientific, San Jose, CA, USA).

A HSS-T3 column, 100 × 2.1 mm, 1.8 μm (Waters, Milford, MA) held at a temperature of 35 °C was used for RP separation. The flow rate was set to 0.3 mL/min for the first 7.5 min and then increased at 0.4 mL/min for the rest of the chromatographic gradient. Mobile phases were 0.1% (v/v) formic acid in water (phase A) and 0.1% (v/v) formic acid in acetonitrile (phase B). The elution gradient had a duration of 20 min and was structured as follows: phase B was held at 0% in the first 3.5 min, then increased to 10% in 4 min and to 35% in 5 min, subsequently from 12.5 to 16 min phase B was held constant at 98%. Finally, the column was returned to the original conditions in 0.5 min and kept stable for 3.5 min to enable its equilibration.

The injection volume was 2 µL. During LC–MS analysis samples were kept in the autosampler at 8 °C.

HILIC separation was achieved with a Acquity UPLC BEH HILIC 2.1 × 100 mm, 1.7 μm, held at a temperature of 35 °C and a constant flow rate of 0.3 mL/min. The mobile phases used were: 20 mM ammonium formate + 0.1% FA at pH 3.7 (phase A) and 0.1% (v/v) formic acid in acetonitrile (phase B). Gradient elution was as follows: linear increase of phase A from 5 to 25% in 8.5 min, followed by a further increase to 60% in 1 min. Phase A was then held constant for 1.5 min and finally returned to 5% in 0.5 min to enable column equilibration for 3.5 min. The total run time was 15 min.

##### Mass spectrometry

Detection was performed on a Q-Exactive Orbitrap MS from Thermo Fisher Scientific (Bremen, Germany) equipped with a heated electrospray ionization (H-ESI). The ionisation was performed in both positive (HESI+) and negative (HESI−) mode, in separate runs. Source conditions were settled as follow: 3.20 kV (pos) / − 3.20 kV (neg) electrospray voltage, capillary temperature 350 °C, S-lens RF level set at 50 (pos) / − 50 (neg), sheath gas (N_2_) flow 40 au, aux gas (N_2_) flow 15 au, where the gas temperature was 350 °C.

All samples, blanks and QCs were acquired in full scan, additionally, pooled QCs were also acquired in triplicate in data dependent MS/MS scan in both positive and negative ion modes. Full scan was carried out with a resolution of 70,000 (FWHM) in a scan range of 50–650 m/z. MS/MS scans were carried out with a stepped normalized collision energy (NCE) of 20 and 50 (eV), and a resolution of 17,500 (FWHM). Three different runs were acquired with different acquisition windows: 50–200 m/z, 200–400 m/z and 400–650 m/z respectively.

All data were acquired in profile mode using Xcalibur^™^ 4.1.

The Q Exactive^™^ mass spectrometer was calibrated for positive and negative mode before sample analysis using the calibration solution provided by the manufacturer (Pierce LTQ ESI positive calibration solution and Pierce LTQ ESI negative calibration solution). For mass-calibration of the instrument a custom list which included lower masses (homovanillic acid, for negative calibration, m/z 181.0500, and sodium fluoroacetate, in positive mode, 101.0015 m/z) as well as the default calibration masses were used to ensure that accurate masses were detected also for low molecular weights compounds^62^.

#### Data Analysis

##### Raw data processing

The raw files obtained in positive and negative ion mode were processed separately using Compound Discoverer^™^ 3.1 (Thermo Scientific^™^, USA) using a non-targeted metabolomics workflow, for retention time alignment, component detection, elemental composition prediction, gap filling. In detail, for peak picking, an adaptive curve algorithm, a resolution of 5 ppm and a maximum retention time shift of 0.5 min were used. Unknown compounds were detected with a mass tolerance of 5 ppm, the S/N threshold was set at 10, relative intensity tolerance for isotope detection was 30%; the minimum peak intensity was set at 10,000. Compounds grouping was achieved with a mass tolerance of 5 ppm and a retention time tolerance of 0.2 min. Blank samples were injected for background subtraction and noise removal during the pre-processing phase; features were filtered out if they appeared in the blank or if they were detected in less than 30% of the QCs and/or with a relative standard deviation (%RSD) greater than 30%.

An output table including m/z versus retention time versus raw peak intensity for all the analysed samples was generated. In addition, normalized area was provided for each detected metabolic feature, by normalization to the pooled QC. The data matrix generated by Compound Discoverer was downloaded as .xls file and two filtration strategies were serially applied to reduce the final number of hits undergoing statistical evaluation. A dilution/filtration strategy^23^ was firstly used by correlating the signal intensity obtained for the diluted QCs to the dilution factor. For each detected metabolic feature, CV% calculated for the diluted samples was required to be < 30%, while the cut-off established for the correlation coefficient R^2^ was 0.8. A further filtration of the data allowed the clusterization of the ions generated from the same parent (adducts, dimers, fragments, isotopes) based on Pearson’s correlation of their intensity. To achieve clusterization, features were sorted by intensity and increasing retention time (Rt); Pearson’s coefficient was calculated for each pair of features eluting at the same Rt (tolerance 0.1 min) and pair of features with Pearson’s coefficient > 0.8 were grouped together (the feature with the largest area was maintained while the other was filtered out).

All four data sets (RP HESI+ , RP HESI− , HILIC HESI+ , HILIC HESI−) were normalised after the acquisition by probabilistic quotient normalization (PQN)^63^ in order to correct differences in urine dilution.

##### Multivariate and univariate statistical analysis

The output table containing filtered metabolic features was exported to Simca^®^ 17 (Umetrics, Umea, Sweden); data were mean centered and Pareto scaled for multivariate analysis by principal component analysis (PCA) to identify outliers (samples which are extremely different from the rest of the data set). Afterwards, to identify differences between specific sample groups (i.e. vehicle vs treated mice) orthogonal partial least squares discriminant analysis (OPLS-DA) as a supervised multivariate approach was used to study the contribution of the variables to groups separation. Validity of the obtained OPLS-DA model and its ability to predict class membership was evaluated based on R^2^Y (> 0.5), Q^2^ (> 0.5), CVAnova (p < 0.05) permutations testing (n = 100) and VIP values (> 1.0). The S-plots were used to highlight the metabolic features with the greatest influence on the separation between groups; the ions of interest to be at different levels between the sample groups were analysed using a paired T-test (GraphPAD Prism). In a following step, with the aim of reducing inter-individual variability and highlight sex differences in metabolome alterations, each mouse was used as its own control by subtracting for each compound the normalized peak area in the vehicle sample from the normalized peak areas in the same animal after drug administration. PCA was used to elucidate how differences like sex, or collection time, could alter the metabolomic fingerprint.

#### Feature identification

Relevant features were searched on mzCloud, ChemSpider as well as in the online database Human metabolome database^64^. A putative identification was achieved upon physicochemical properties (monoisotopic exact mass, isotopic pattern) and by correspondence with mass spectra (MS/MS fragments) of available libraries.

## Conclusions

In this study untargeted metabolomics was exploited to investigate changes in the urinary endogenous metabolite levels in murine models, following both natural and synthetic opioids administration. A strength of the study is the inclusion of animals of both sex in order to investigate sexual diergism in the metabolic changes associated with opioid use. According to our results, the intake of these drugs resulted in an elevated energy demand, especially following fentanyl administration, and it was more pronounced on male animals, as evidenced by the increase in medium and long chain acylcarnitines levels. It was also shown that opioid administration disrupts the pathways related to catecholamines biosynthesis characterising the effect of these drugs on CNS function. The observed alterations were common to both morphine and fentanyl, suggesting that they are not related to the chemical structure of the drug, but rather on the drug class, highlighting the potential of metabolomics in forensic toxicology for investigations related to NSOs or more generally NPS. Moreover, this study shows that opioids modulate metabolic pathways in male and female subjects differently although they cause the same acute analgesic effects (positive effects in therapy) and respiratory depression (negative effects). This underlines the importance of investigating carefully and with new technologies the effects of NPS on gender difference. Based on previous metabolomics studies, related to other drugs of abuse, the observed metabolic signature is not exclusive to opioid usage, nevertheless, the findings discussed herein lay the groundwork for subsequent investigations, involving different classes of psychoactive drugs, that can help to identify distinctive biomarkers of drug class use.

As usual with metabolomic studies through HRMS, limitations to our results are related to the uncomplete annotation of the significantly altered features as well as the lack of authentic standards to confirm the identity of the metabolites, however it must be pointed out that only the features with almost perfect MS/MS spectra matching were discussed and reported in the tables.

Furthermore, it would be interesting to study the variation of the urinary metabolic profile also by inducing abstinence to verify the return of biomarkers to normal pre-treatment levels.

## Data Availability

The datasets generated during and/or analysed during the current study are available from the corresponding author on reasonable request.
